# New Neuromuscular Training for Peripheral Nerve Disorders Using an Ankle Joint Hybrid Assistive Limb: A Case Series

**DOI:** 10.3390/medicina59071251

**Published:** 2023-07-05

**Authors:** Yuichiro Soma, Kunihiko Tokunaga, Shigeki Kubota, Mikio Muraoka, Shin Watanabe, Michiko Sakai, Wataru Ohya, Daiki Arakawa, Takuma Sasage, Masashi Yamazaki

**Affiliations:** 1 Department of Rehabilitation Medicine, Institute of Medicine, University of Tsukuba, Tsukuba 305-8577, Japan; 2 Niigata Hip Joint Center, Kameda Daiichi Hospital, Niigata 950-0165, Japan; 3 Department of Orthopaedic Surgery, Institute of Medicine, University of Tsukuba, Tsukuba 305-8577, Japanmasashiy@md.tsukuba.ac.jp (M.Y.); 4 Department of Orthopaedic Surgery, Kameda Daiichi Hospital, Niigata 950-0165, Japan; 5 Department of Electrophysiolosical Studies, Kameda Daiichi Hospital, Niigata 950-0165, Japan; 6 Department of Rehabilitation, Kameda Daiichi Hospital, Niigata 950-0165, Japanhpa12008@nuhw.ac.jp (D.A.);

**Keywords:** robotic ankle rehabilitation, neuromuscular training, peripheral nerve disorder, feedback motor learning, muscle action potential

## Abstract

Peripheral nerve disorder of the lower extremities causes drop foot and disturbs the daily living activities of patients. The ankle joint hybrid assistive limb (HAL) provides voluntary ankle joint training using surface bioelectrical signals from the muscles of the lower extremities. We investigated the neurological effects of ankle joint HAL training in three patients. Sensory nerve action potentials (SNAPs) and compound muscle action potentials (CMAPs) were analyzed for the peroneal and tibial nerves prior to the first ankle joint HAL training session. Integrated surface electromyography EMG signals were recorded before and after the HAL training sessions to evaluate the effects of training for neuromuscular disorders. The patients were hospitalized to receive rehabilitation with HAL training for 2 weeks. The HAL training was performed daily with two 60 min sessions. All cases demonstrated severe neuromuscular impairment according to the result of the CMAP. All integrated EMG measurements of antagonistic muscle activities decreased after the ankle joint HAL training. The manual muscle testing (MMT) scores of each muscle were slightly increased after the HAL intervention for Case 2(tibialis anterior, from 2 to 2+; gastrocnemius muscles, from 2− to 2; extensor digitorum longus, and extensor hallucis longus, from 1 to 3). The MMT scores were also slightly increased except for gastrocnemius muscle for Case 3 (tibialis anterior, extensor digitorum longus, and extensor hallucis longus, from 2− to 2). These two patients demonstrated voluntary muscle contractions and nerve signals in the CMAP before the HAL training. Even though the amplitude of CMAPs was low, the HAL training may provide voluntary ankle joint movements by reducing the antagonistic muscle contraction via computer processing. The HAL training may enhance muscle movement and coordination through motor learning feedback.

## 1. Introduction

Iatrogenic nerve injuries can occur due to compression, traction, ischemia, and traction during some orthopedic surgeries [1]. Peripheral nerve injuries typically involve mixed nerves with both sensory and motor components. Damage to either of these nerves can result in sensory alterations and weakness in the lower limb. Recovery from nerve palsy can be very slow and incomplete [2]. Nerve injury may occur anywhere from the sciatic origin to the terminal branches in the foot and ankle [3]. Complete neural recovery of sciatic nerve palsy occurs in only 16~36% of patients [4,5]. Peroneal nerve palsy often occurs with entrapment neuropathies of the lower extremities, which can result in drop foot and can disturb patients’ daily living [6,7]. The establishment of an effective conservative treatment for drop foot resulting from peroneal nerve palsy remains elusive. Some reports have suggested the efficacy of functional electrical stimulation as a conservative treatment for managing drop foot [8,9]. However, functional electrical stimulation treatment has not gained widespread acceptance in clinical settings, limiting its status as an established treatment method. While orthotic treatment [10] can effectively prevent ankle joint deformity and serve as an assistive walking device, it does not address the underlying palsy. Consequently, the establishment of an effective conservative treatment for drop foot, which can arise from various factors such as peroneal neuropathy at the neck of the fibula, L5 radiculopathy, or stroke, is still lacking. Therefore, effective neurorehabilitation is essential for patients with sciatic or peroneal nerve palsies. To our knowledge, there are few studies investigating effective neural treatment using feedback learning for patients with lower extremity nerve palsy. The ankle HAL is a wearable exoskeleton-type robot that is used to train plantar and dorsiflexion and for voluntary assistive training of the ankle joint of patients with palsy using an actuator, which is placed on the lateral side of the ankle joint and detects bioelectrical signals (muscle action potentials) from the tibialis anterior (TA) and gastrocnemius muscles. The HAL provides the wearer with a feedback system that allows the wearer to observe their own bioelectrical signals in real time on a monitor during ankle HAL training. Voluntary active ankle dorsiflexion training using the ankle HAL is crucial for errorless learning [11] and motor learning [12] in the field of neurorehabilitation. For motor learning, training that involves active participation and voluntary movement production by the subject is considered essential for inducing changes in motor performance, cortical activity, and excitability [13]. Another important factor in motor learning is providing correct afferent input. The HAL training can provide the correct motion for paralytic ankle dorsiflexion despite low and weak muscle activity and can change muscle EMG waves during active movement. In our previous study, we conducted HAL training using the novel robotics ankle HAL and demonstrated its safety, feasibility, and effectiveness as a treatment for a patient with ankle-foot disability resulting from L5 palsy after lumbar spine surgery [14]. However, it is important to note that the study was limited to a single subject, and we did not specifically evaluate nerve conduction or EMG in that study. Our hypothesis is that ankle joint HAL training might help optimize muscle activities, which may enhance coordination by the inhibition or reduction in antagonist muscle activity. In this study, we treated three patients with L5, S1 palsy or peroneal, and tibial nerve palsy using ankle joint HAL training and assessed nerve conduction as well as integrated EMGs.

## 2. Materials and Methods

### 2.1. Patients

Three patients were included in this study. This case study focuses on the severity of peripheral neuropathy. Case 1 was an 85-year-old woman diagnosed with lumbar disc herniation at the L4/5 level. She had left peroneal nerve palsy for more than 20 years since the onset. Case 2 was a 58-year-old woman diagnosed with degenerative lumbar spondylolisthesis. She had experienced sciatic nerve palsy for 6 years since the onset. Case 3 was a 71-year-old man diagnosed with left peroneal nerve palsy due to an ischemic injury after total knee arthroplasty, and it had been 7 months since the onset. Patients’ clinical data are shown in Table 1.

### 2.2. Ankle Joint HAL Training

**Figure 1:**
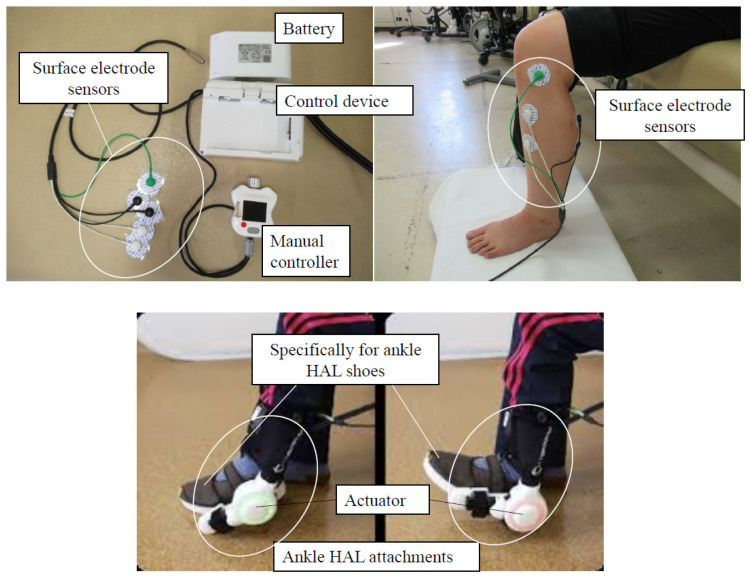
The structure of the ankle HAL consists of a control device, battery, manual controller, surface electrode sensors, specifically for the ankle HAL shoe, an actuator, and an ankle HAL attachment.

The HAL can enhance movement by expressing the wearer’s voluntary muscle activity as actual motion. Through repeated execution of specific tasks, it promotes the reinstatement or restructuring of appropriate proprioceptive feedback learning, which is expected to improve the wearer’s operational capacity. These HAL motion support technologies incorporate an interactive biofeedback system that links the brain/nervous system body to the HAL [15]. The ankle joint HAL system consists of a control device, battery, manual controller, surface electrode sensors, specifically for the ankle HAL shoe, an actuator, and an ankle HAL attachment (Figure 1).

**Figure 2:**
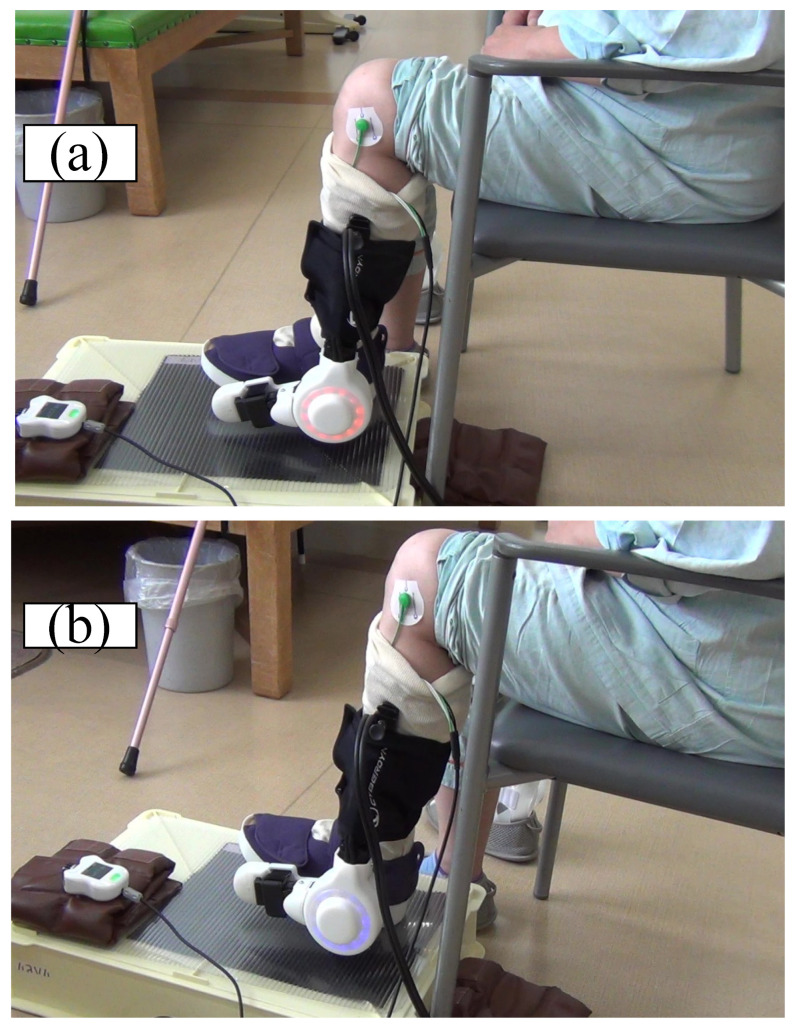
(**a**) Resting position of the ankle HAL; (**b**) Maximum left ankle dorsiflexion with the ankle HAL.

The ankle joint HAL can detect bioelectrical signals from the tibialis anterior muscle to promote dorsiflexion in patients with sciatic or peroneal nerve palsies. First, the patient was placed in a seated position and the therapist attached the ankle HAL. The surface electrode sensors of the HAL were then attached to the tibialis anterior and gastrocnemius muscles and the sensor cables were plugged into the control device. Subsequently, the muscle activities of the tibialis anterior and gastrocnemius muscles were observed on the manual controller monitor. The torque of the ankle HAL actuator was then adjusted and appropriately positioned at a dorsiflexed angle during the ankle HAL training [14]. The patients were hospitalized to receive rehabilitation with HAL training for 2 weeks. The HAL training was performed daily with two 60 min sessions (Figure 2). Concurrently, all patients received conventional rehabilitation, which included passive range of motion and muscle strengthening training around the hip and knee joints by physical therapists.

### 2.3. Measurements

We performed manual muscle testing (MMT) of the tibialis anterior, extensor hallucis longus, extensor digitorum longus, and gastrocnemius muscles to assess the improvement in muscle strength after HAL training.

Nerve conduction studies are believed to be the most accurate, reliable, and sensitive measurement of peripheral nerve function. In comparison to nerve biopsy, nerve conduction studies is a noninvasive procedure, making it the preferred choice for evaluating peripheral nerve disorders [16]. The purpose of conducting nerve conduction studies in this study was to assess the severity of peripheral neuropathy, particularly in cases where the use of HAL is indicated. The objective was to evaluate the extent of nerve fiber loss and identify any signs of recovery in each individual case. Nerve conduction studies were performed on the peroneal and tibial nerves before the first ankle joint HAL training. We assessed the amplitude of the SNAPs and CMAPs to evaluate the severity of axonal loss [17] as a measure of peripheral nerve disorder [18]. The peroneal nerves were stimulated supra-maximally at three sites, including the popliteal fossa, fibular head, and dorsal ankle. The CMAPs of the extensor digitorum brevis (EBD) were detected. The superficial peroneal nerves were antidromically stimulated above the ankle. The tibial nerves were also supra-maximally stimulated at the popliteal fossa and tarsal tunnel and the CMAPs of the abductor hallucis (AH) were detected. The medial plantar nerves were orthodromically stimulated via the thenar muscles.

We recorded the integrated surface EMG signals before and after the HAL training sessions. Muscle activities were recorded from the tibialis anterior and gastrocnemius muscles. The EMG electrodes were placed on the tibialis anterior and gastrocnemius muscles. Electrode placement on the examined muscles was based on the SENIAM recommendations [19]. The surface EMGs were obtained by directing the patients to maintain the ankle joint in a maximum dorsal and plantar flexion for 20 s. All EMG signals were filtered with a band-pass filter (2–20 kHz). The data were rectified and integrated to obtain an integrated EMG profile over 10 s. The nerve conduction study and surface EMGs were performed using Neuropack (MEB2200, Nihon Kohden, Tokyo, Japan).

## 3. Results

Table 2 shows the MMT scores before and after the ankle joint HAL training sessions. In Case 2, the MMT scores of each muscle were increased slightly after the HAL interventions (tibialis anterior, from 2 to 2+; gastrocnemius muscles, from 2− to 2; extensor digitorum longus, and extensor hallucis longus, from 1 to 3). A video recording demonstrates the autonomic movements of the ankle and toes in Case 2 before and after the ankle joint HAL training sessions (Video S1). Ankle dorsiflexion and toe extension were improved in agreement with the increased MMT scores.

In Case 3, the MMT scores of the tibialis anterior, extensor digitorum longus, and extensor hallucis longus muscles were increased slightly except for the MMT of the gastrocnemius muscle (tibialis anterior, extensor digitorum longus and extensor hallucis longus, from 2− to 2). However, all MMT scores in Case 1 remained changed.

The amplitudes of the SNAPs and CMAPs are shown in Table 3.

Both amplitudes from the SNAP and CMAP of the affected limbs were low for Case 1. The superficial peroneal nerve-SNAP and EDB-CMAP were not detected, the amplitude of AH-CMAP was much lower, and the medial plantar nerve-SNAP was undetected in Case 2.

In Case 3, the amplitude of the EDB-CMAP on the affected side was very low, and the SNAP of the peroneal and tibial nerves was not detected on the affected side.

The integrated EMG of the tibialis anterior and gastrocnemius muscles are shown before and after the ankle joint HAL training sessions in Table 4. All integrated EMG measurements after the HAL training sessions decreased after training.

## 4. Discussion

We investigated the neurophysiological effects of the ankle joint HAL training for peripheral nerve disorders on lower extremities using nerve conduction and surface EMG.

The amplitudes of the CMAPs demonstrated severe neuropathies in all cases. In addition, all patients’ MMT scores were either not increased or slightly increased after the ankle joint HAL sessions. In contrast, all integrated EMGs after the HAL sessions were decreased after training. Ankle dorsiflexion and toe extension were improved in agreement with the slightly increased MMT scores in Case 2. These muscle relaxations may inhibit the abnormal EMG antagonistic muscle activation during the ankle and toe joint movements [20]. A previous study showed that spastic co-contraction corresponds to excessive activity of the antagonist muscles during active movement [21]. Another study highlighted the relationship between spastic co-contraction and the range of motion restriction [22]. Hence, the ankle joint HAL training decreased antagonist muscle activation resulting in a relatively increased agonist muscle activation and a slightly increased MMT.

The patients had the potential for voluntary muscle contraction of the EDB or AH muscles, and the nerve signals were quite small according to the CMAPs. Even if the surface muscle action potentials were small, the ankle joint HAL could generate voluntary ankle joint movements, and thus provide a proprioceptive feedback loop [23]. In spite of the voluntary muscle contractions, the nerve signals from the SNAP and CMAP (SNAP; 8.9 μv, CMAP; 190 μv), and the increased muscle activities on the EMG, muscle strength was not improved in Case 1. The onset of the nerve palsy in Case 1 was 20 years prior, for which the neural functional recovery may have reached a plateau status. In addition, joint contracture hampered the subject’s ankle movement. In Case 1, another important factor that we considered is spasticity, which is defined as a motor disorder characterized by increased muscle tone and resistance to passive movement. Spasticity can significantly hinder the initiation of active rehabilitation [24]. In contrast, the ankle joint HAL training provided effective treatment for Cases 2 and 3, who both had symptom onset within a relatively short period. The effects of ankle-foot orthosis were reported, indicating that individuals without a contracture benefit from an ankle-foot orthosis that allows for dorsiflexion mobility [10]. In our previous study, we demonstrated that improvement in passive dorsiflexion ROM was observed after ankle HAL training [14]. The previous study showed that changes in muscle EMG waves of the tibialis anterior muscles were caused by an increase in voluntary movements, suggesting that patients’ voluntary movement of the tibialis anterior muscles was accelerated by the ankle joint HAL training [14]. In this study, the HAL training altered the involuntary EMG waves as well as the voluntary EMG waves by reducing antagonistic muscle contraction with computer processing [25,26]. However, the ankle joint HAL training might not affect recovery from peripheral nerve disorder when the neural recovery has reached a plateau status. Neural activity and repeated execution of specific tasks promote the reinstatement or restructuring of appropriate proprioceptive feedback learning [27]. Therefore, the ankle joint HAL training enhanced the muscle movement or coordination of patients who had symptom onset within a relatively short period by feedback motor learning [14]. There were some limitations in this study. We investigated only surface EMG measurements to evaluate the effect of the ankle joint HAL training. In addition, nerve conduction studies were performed just before the first ankle joint HAL training session. Therefore, it was difficult to distinguish what was the most effective factor to improve neuromuscular impairment by the HAL training. Nevertheless, we recognize that there are alternative assessments available for evaluating peripheral nerve function, such as ultrasound imaging [28]. Furthermore, the ankle joint HAL training protocol was possibly unsuitable for Case 1, who had a long period since onset. Future studies are required with a greater number of patients performing a statistical analysis of intervention data and a control group to explore the specific protocol for the effective treatment of nerve palsy by ankle joint HAL training. Additionally, it is important to include other measurements in our study, such as passive and active ROM or assessments of spasticity. These additional measurements will provide a more comprehensive evaluation of the outcomes and factors affecting the effectiveness of our intervention.

## 5. Conclusions

Even if the patients demonstrated only minimal muscle action potentials by electrophysiological examination, the ankle joint HAL training enhanced muscle movement and coordination of patients who had symptom onset within a relatively short period by feedback motor learning. The ankle joint HAL could be an effective neural treatment to reinstate movement from feedback learning for patients with nerve palsies of the lower extremities.

## Figures and Tables

**Table 1:** Clinical characteristics of the patients.

Case	1	2	3
Sex	Female	Female	Male
Age	85	58	71
Post onset period	20 years	6 years	7 months
Diagnosis	Lumbar disc herniation, LCS	Degenerative lumbar spondylolisthesis	Ischemia after total knee arthroplasty
Type of paralysis	L5 palsy	L5, S1 palsy	Peroneal nerve palsy Tibial nerve palsy

LCS, lumbar canal stenosis.

**Table 2:** MMT scores before and after the ankle HAL training sessions.

		Tibialis Anterior	Gastrocnemius Muscles	EHL	EDL
Case 1	Pre-HAL	2	5	2	2
	Post-HAL	2	5	2	2
Case 2	Pre-HAL	2	2-	1	1
	Post-HAL	2+	2	3	3
Case 3	Pre-HAL	2-	2+	2-	2-
	Post-HAL	2	2+	2	2

Pre-HAL, before the first ankle HAL training session; Post-HAL, after all of the ankle HAL training sessions; EHL, extensor hallucis longus; EDL, extensor digitorum longus.

**Table 3:** The amplitude of the CMAPs and SNAPs before the ankle joint HAL intervention.

Case		1	2	3
Peroneal nerve	CMAP	190 μv	(-)	780 μv
	SNAP	8.9 μv	(-)	(-)
Tibial nerve	CMAP	7.1 mv	0.2 mv	4.5 mv
	SNAP	1.9 μv	(-)	(-)

CMAP, compound muscle action potential; SNAP, sensory nerve action potential; HAL, hybrid assistive limb; (-), undetected; The peroneal nerve palsy was measured by the extensor digitorum brevis of the CMAP and the amplitude of the superficial peroneal nerve of the SNAP. The tibial nerve palsy was measured by the abductor hallucius of the CMAP and the amplitude of the medial plantar nerve of the SNAP.

**Table 4:** Integrated EMGs of the tibialis anterior and gastrocnemius muscles (mVms).

		Dorsiflexion		Plantar Flexion	
		Tibialis Anterior	Gastrocnemius	Tibialis Anterior	Gastrocnemius
Case 1	Pre-HAL	201	176	583	3613
	Post-HAL	115	170	494	2253
Case 2	Pre-HAL	189	240	241	1994
	Post-HAL	132	229	229	1122
Case 3	Pre-HAL	969	401	334	857
	Post-HAL	650	289	311	447

Pre-HAL, before the first ankle HAL training session; Post-HAL, after all of the ankle HAL training sessions.

## Supplementary Materials

The following supporting information can be downloaded at: https://www.mdpi.com/article/10.3390/medicina59071251/s1, Video S1: The video documents the movement of the ankle and toe joints in Case 2 before and after the ankle joint HAL training session at 2 days and 1 month post training.

## References

[1] Hasija R., Kelly J.J., Shah N.V., Newman J.M., Chan J.J., Robinson J., Maheshwari A.V. (2018). Nerve injuries associated with total hip arthroplasty. J. Clin. Orthop. Trauma.

[2] Haddad F.S. (2017). Retraction: Post-operative neuropathy after total hip arthroplasty. Bone Jt. J..

[3] Poage C., Roth C., Scott B. (2016). Peroneal Nerve Palsy: Evaluation and Management1. Am. Acad. Orthop. Surg..

[4] Edwards B.N., Tullos H.S., Noble P.C. (1987). Contributory factors and etiology of sciatic nerve palsy in total hip arthroplasty. Clin. Orthop. Relat. Res..

[5] Farrell C.M., Springer B.D., Haidukewych G.J., Morrey B.F. (2005). Motor nerve palsy following primary total hip arthroplasty. J. Bone Jt. Surg. Am..

[6] Carolus A.E., Becker M., Cuny J., Smektala R., Schmieder K., Brenke C. (2019). The interdisciplinary management of foot drop. Dtsch. Ärzteblatt Int..

[7] Lamontagne A., Malouin F., Richards C., Dumas F. (2002). Mechanisms of disturbed motor control in ankle weakness during gait after stroke. Gait Posture.

[8] Kafri M., Laufer Y. (2014). Therapeutic effects of functional electrical stimulation on gait in individuals post-stroke. Ann. Biomed. Eng..

[9] Bosch P.R., Harris J.E., Wing K. (2014). American Congress of Rehabilitation Medicine (ACRM) Stroke Movement Interventions Subcommittee. Review of therapeutic electrical stimulation for dorsiflexion assist and orthotic substitution from the american congress of rehabilitation medicine stroke movement interventions subcommittee. Arch. Phys. Med. Rehabil..

[10] Mulroy S.J., Eberly V.J., Gronely J.K., Weiss W., Newsam C.J. (2010). Effect of AFO design on walking after stroke: Impact of ankle plantar flexion contracture. Prosthet. Orthot. Int..

[11] Radulescu A., Cox K., Adams P. (2009). Hebbian errors in learning: An analysis using the Oja model. J. Theor. Biol..

[12] Pennycott A., Wyss D., Vallery H., Klamroth-Marganska V., Riener R. (2012). Towards more effective robotic gait training for stroke rehabilitation: A review. J. Neuroeng. Rehabil..

[13] Lotze M., Braun C., Birbaumer N., Anders S., Cohen L. (2003). Motor learning elicited by voluntary drive. Brain.

[14] Kubota S., Kadone H., Shimizu Y., Koda M., Noguchi H., Takahashi H., Watanabe H., Hada Y., Sankai Y., Yamazaki M. (2022). Development of a New Ankle Joint Hybrid Assistive Limb. Medicina.

[15] Kubota S., Abe T., Kadone H., Shimizu Y., Funayama T., Watanabe H., Marushima A., Koda M., Hada Y., Sankai Y. (2019). Hybrid assistive limb (HAL) treatment for patients with severe thoracic myelopathy due to ossification of the posterior longitudinal ligament (OPLL) in the postoperative acute/subacute phase: A clinical trial. J. Spinal Cord Med..

[16] Nasseri K., Strijers R.L., Dekhuijzen L.S., Buster M., Bertelsmann F.W. (1998). Reproducibility of different methods for diagnosing and monitoring diabetic neuropathy. Electromyogr. Clin. Neurophysiol..

[17] Thaisetthawatkul P., Logigian E., Herrmann D. (2002). Dispersion of the distal compound muscle action potential as a diagnostic criterion for chronic inflammatory demyelinating polyneuropathy. Neurology.

[18] Falco F.J.E., Hennessey W.J., Braddom R.L., Goldberg G. (1992). Standardized Nerve conduction studies in the Upper Limb of the healthy elderly. Am. J. Phys. Med. Rehabil..

[19] Hermens H.J., Freriks B., Disselhorst-Klug C., Rau G. (2000). Development of recommendations for SEMG sensors and sensor placement procedures. J. Electromyogr. Kinesiol..

[20] Banks C.L., Huang H.J., Little V.L., Patten C. (2017). Electromyography exposes heterogeneity in muscle co-contraction following stroke. Front. Neurol..

[21] Soma Y., Mutsuzaki H., Yoshioka T., Kubota S., Shimizu Y., Kanamori A., Yamazaki M. (2022). Single-joint Hybrid Assistive Limb in Knee Rehabilitation after ACL Reconstruction: An Open-label Feasibility and Safety Trial. Prog. Rehabil. Med..

[22] Chalard A., Amarantini D., Tisseyre J., Marque P., Tallet J., Gasq D. (2019). Spastic co- contraction, rather that spasticity, is associated with impaired active function in adults with acquired brain injury: A pilot study. J. Rehabil. Med..

[23] Sczesny-Kaiser M., Höffken O., Aach M., Cruciger O., Grasmücke D., Meindl R., Schildhauer T.A., Schwenkreis P., Tegenthoff M. (2015). HAL® exoskeleton training improves walking parameters and normalizes cortical excitability in primary somatosensory cortex in spinal cord injury patients. J. Neuroeng. Rehabil..

[24] Ikumi A., Kubota S., Shimizu Y., Kadone H., Marushima A., Ueno T., Kawamoto H., Hada Y., Matsumura A., Sankai Y. (2017). Decrease of spasticity after hybrid assistive limb® training for a patient with C4 quadriplegia due to chronic SCI. J. Spinal Cord. Med..

[25] Kubota S., Abe T., Koda M., Kadone H., Shimizu Y., Mataki Y., Noguchi H., Fujii K., Marushima A., Funayama T. (2018). Application of a newly developed upper limb single-joint hybrid assistive limb for postoperative C5 paralysis: An initial case report indicating its safety and feasibility. J. Clin. Neurosci..

[26] Suzuki K., Gouji M., Kawamoto H., Hasegawal Y., Sankai K. (2007). Intention-based walking support for paraplegia patients with robot suit HAL. Adv. Robot..

[27] Chisholm A.E., Alamro R.A., Williams A.M., Lam T. (2017). Overground vs. treadmill-based robotic gait training to 422 improve seated balance in people with motor-complete spinal cord injury: A case report. J. Neuroeng. Rehabil..

[28] Boon A. (2013). Ultrasonography and Electrodiagnosis: Are They Complementary Techniques?. PM&R.

